# Blastocyst telomere length predicts successful implantation after frozen-thawed embryo transfer

**DOI:** 10.1093/hropen/hoae012

**Published:** 2024-02-24

**Authors:** Chun-Wei Chien, Yen-An Tang, Shuen-Lin Jeng, Hsien-An Pan, H Sunny Sun

**Affiliations:** Center for Genomic Medicine, Innovation Headquarters, National Cheng Kung University, Tainan, Taiwan; Center for Genomic Medicine, Innovation Headquarters, National Cheng Kung University, Tainan, Taiwan; Institute of Molecular Medicine, College of Medicine, National Cheng Kung University, Tainan, Taiwan; Department of Statistics, Institute of Data Science, National Cheng Kung University, Tainan, Taiwan; Center for Innovative FinTech Business Models, National Cheng Kung University, Tainan, Taiwan; IVF center, An-An Women and Children Clinic, Tainan, Taiwan; Department of Obstetrics and Gynecology, College of Medicine, National Cheng Kung University, Tainan, Taiwan; Center for Genomic Medicine, Innovation Headquarters, National Cheng Kung University, Tainan, Taiwan; Institute of Molecular Medicine, College of Medicine, National Cheng Kung University, Tainan, Taiwan

**Keywords:** assisted reproduction, embryo development, embryo transfer, genetic diagnosis, implantation, infertility, IVF/ICSI outcome, PGS

## Abstract

**STUDY QUESTION:**

Do embryos with longer telomere length (TL) at the blastocyst stage have a higher capacity to survive after frozen-thawed embryo transfer (FET)?

**SUMMARY ANSWER:**

Digitally estimated TL using low-pass whole genome sequencing (WGS) data from the preimplantation genetic testing for aneuploidy (PGT-A) process demonstrates that blastocyst TL is the most essential factor associated with likelihood of implantation.

**WHAT IS KNOWN ALREADY:**

The lifetime TL is established in the early cleavage cycles following fertilization through a recombination-based lengthening mechanism and starts erosion beyond the blastocyst stage. In addition, a telomerase-mediated slow erosion of TL in human fetuses has been observed from a gestational age of 6–11 weeks. Finally, an abnormal shortening of telomeres is likely involved in embryo loss during early development.

**STUDY DESIGN, SIZE, DURATION:**

Blastocyst samples were obtained from patients who underwent PGT-A and FET in an IVF center from March 2015 to May 2018. Digitally estimated mitochondrial copy number (mtCN) and TL were used to study associations with the implantation potential of each embryo.

**PARTICIPANTS/MATERIALS, SETTING, AND METHODS:**

In total, 965 blastocysts from 232 cycles (164 patients) were available to investigate the biological and clinical relevance of TL. A WGS-based workflow was applied to determine the ploidy of each embryo. Data from low-pass WGS-PGT-A were used to estimate the mtCN and TL for each embryo. Single-variant and multi-variant logistic regression, decision tree, and random forest models were applied to study various factors in association with the implantation potential of each embryo.

**MAIN RESULTS AND THE ROLE OF CHANCE:**

Of the 965 blastocysts originally available, only 216 underwent FET. While mtCN from the transferred embryos is significantly associated with the ploidy call of each embryo, mtCN has no role in impacting IVF outcomes after an embryo transfer in these women. The results indicate that mtCN is a marker of embryo aneuploidy. On the other hand, digitally estimated TL is the most prominent univariant factor and showed a significant positive association with pregnancy outcomes (*P* < 0.01, odds ratio 79.1). We combined several maternal and embryo parameters to study the joint effects on successful implantation. The machine learning models, namely decision tree and random forest, were trained and yielded classification accuracy of 0.82 and 0.91, respectively. Taken together, these results support the vital role of TL in governing implantation potential, perhaps through the ability to control embryo survival after transfer.

**LIMITATIONS, REASONS FOR CAUTION:**

The small sample size limits our study as only 216 blastocysts were transferred. The number was further reduced to 153 blastocysts, where pregnancy outcomes could be accurately traced. The other limitation of this study is that all data were collected from a single IVF center. The uniform and controlled operation of IVF cycles in a single center may cause selection bias.

**WIDER IMPLICATIONS OF THE FINDINGS:**

We present novel findings to show that digitally estimated TL at the blastocyst stage is a predictor of pregnancy capacity after a FET cycle. As elective single-embryo transfer has become the mainstream direction in reproductive medicine, prioritizing embryos based on their implantation potential is crucial for clinical infertility treatment in order to reduce twin pregnancy rate and the time to pregnancy in an IVF center. The AI-powered, random forest prediction model established in this study thus provides a way to improve clinical practice and optimize the chances for people with fertility problems to achieve parenthood.

**STUDY FUNDING/COMPETING INTEREST(S):**

This study was supported by a grant from the National Science and Technology Council, Taiwan (MOST 108-2321-B-006-013 -). There were no competing interests.

**TRIAL REGISTRATION NUMBER:**

N/A.

WHAT DOES THIS MEAN FOR PATIENTS? Telomeres are stretches of DNA found at the ends of the chromosomes. They cap and protect the end of a chromosome like the end of a shoelace. Telomeres are crucial for the survival of all living cells and telomere length (TL) is the key to controlling lifespan and aging of a cell. Previous research hinted at the importance of TL in early human development, suggesting that abnormal shortening may lead to embryo loss and implantation failure. In this study, we directly estimated TL in embryos using sequencing data from preimplantation genetic testing. The study aimed to determine if embryos with longer TL have a higher chance of successful implantation after transfer. Our findings highlight that blastocyst TL is a critical factor influencing implantation potential, likely because of its role in controlling embryo survival after transfer. In an attempt to reduce to time to pregnancy in the *in vitro* fertilization (IVF) processes, we studied various maternal and embryo parameters, including TL, that have a high impact on successful implantation into an artificial intelligence model suitable for routine use in IVF clinics. Prioritizing embryos based on implantation potential is vital in clinical infertility treatment, aiming to reduce twin pregnancies and shorten waiting times during IVF. The predictive model developed in this study offers a valuable tool to enhance clinical practice, providing an optimized approach for individuals facing fertility challenges to increase their chances of achieving parenthood.

## Introduction

Infertility affects millions of people of reproductive age and has become the third most common disease globally after cardiovascular disease and cancer in the 21st century (Akalewold *et al.*, 2022). With help from *in vitro* fertilization (IVF) technology, it is now estimated that more than 6 million babies have been born through the IVF procedure. However, many factors, including maternal dysfunction and embryonic chromosomal abnormalities, can cause the failure of IVF treatment resulting in an overall live birth rate for the first cycle of 29.5% (95% CI: 29.3, 29.7) in the UK (Smith *et al.*, 2015), 32.7% (95% CI: 32.2, 33.1%) in Australia and New Zealand (Chambers *et al.*, 2017), and 32.5% in the USA (National Center for Chronic Disease Prevention and Health Promotion Division of Reproductive Health, Centers for Disease Control and Prevention, 2023). Previously, studies have reported that chromosome integrity (i.e. euploidy) is the primary determinant of IVF success (Tšuiko *et al.*, 2019); thus, with the additional support of preimplantation genetic testing for aneuploidy (PGT-A), the IVF success rates have been improved in women with advanced maternal age and those who experience recurrent miscarriage (RM) (Platteau *et al.*, 2005; Sacchi *et al.*, 2019). Nevertheless, the success rate only rises to 50%. Thus, developing methods which will increase the implantation rate is the top priority of the current move towards personalized maternal–fetal medicine in an IVF center. Furthermore, the results also indicate that, in addition to chromosomal integrity of the embryo, other factors related to embryo viability may need to be considered to maximize the efficacy of IVF treatment.

Mitochondria are involved in the regulation of multiple essential cellular processes, such as apoptosis, amino acid synthesis, calcium homeostasis, and the generation of energy in the form of ATP via the process of oxidative phosphorylation (OXPHOS) (Dumollard *et al.*, 2009; St John *et al.*, 2010; Tilly and Sinclair, 2013). Recent studies showed that higher amounts of mitochondrial DNA (mtDNA) are associated with cellular ‘stressed’ conditions such as aneuploidy, advanced maternal age, or chemically induced stress (Leese, 2002; Diez-Juan *et al.*, 2015; Fragouli *et al.*, 2015). Consequently, studies reported that embryos with mtDNA levels higher than average showed a poor implantation rate (Diez-Juan *et al.*, 2015; Fragouli *et al.*, 2015). However, confirmatory studies from other groups failed to validate the initial findings and suggested that mtDNA cannot predict the reproductive potential of embryos (St John, 2016; Treff *et al.*, 2017; Ritu *et al.*, 2022). Furthermore, a review by Seli has concluded that mtDNA copy number (mtCN) may be used as a biomarker for embryo viability (Seli, 2016). Still, future studies must clarify whether mtCN is an independent biomarker of embryo morphology, and in PGS, and whether its use may result in higher IVF pregnancy rates (Seli, 2016). Therefore, how to correctly evaluate the proportion of mtCN and whether mtCN can be used as a predictive factor for embryo implantation is still controversial.

Telomeres consist of a repeated TTAGGG DNA sequence, which typically extends over several thousand base pairs at chromosome ends, and are known to play vital roles in multiple cellular processes because they protect chromosomes from end-to-end fusions and chromosomal instability (Aksenova and Mirkin, 2019). Previous studies have applied different methodologies to measure telomere length (TL) in various cells and proposed that TL is considered a molecular marker of sperm quality and male infertility (Boniewska-Bernacka *et al.*, 2019), granulosa cells and female fertility (Fattet *et al.*, 2020), and few female clinical conditions (Rocca *et al.*, 2019). Studies have shown that telomere shortening induces apoptosis in human preimplantation embryos (Keefe *et al.*, 2005), and analyses in human and mice strongly support the notion that a minimum TL is likely required for the development of a competent embryo (Kalmbach *et al.*, 2014). TL has been proposed to play an essential role in the development of the embryo and fetus, and the abnormal shortening of telomeres is likely involved in embryo loss during early human development (Kohlrausch *et al.*, 2021). Nevertheless, the TL measured in these studies using either cumulus cells or granulosa cells can only provide an estimate of TL in embryos.

We hypothesized that embryos with longer TL at the blastocyst stage might have a higher capacity to survive after frozen-thawed embryo transfer (FET). To test this hypothesis, we established a next-generation sequencing (NGS)-based approach to estimate TL using low-pass whole genome sequencing (WGS) data from the IVF with PGT-A process. We then applied digital-estimated TL in 965 blastocysts to study the influence of TL on embryo survival and implantation potential following FET. In addition, we developed an artificial intelligence (AI) model using several maternal and embryonic factors, including TL, that could be functional in routine IVF clinics to accelerate the IVF journey.

## Materials and methods

### Patients, standard DNAs, and ethics statement

Patients who underwent IVF and PGT-A in the An-An Women and Children Clinic had signed consent forms for embryo biopsy and aneuploidy screening. When the patients signed the consent forms, they were aware that embryo biopsy and PGT-A are investigational procedures requiring the removal of one or more cells from embryos and that the genetic analysis of their samples would be used for the selection of euploid embryos for transfer and the data would be used for investigation purposes. PGT-A samples were derived from trophectoderm (TE) biopsies (typically 5–10 cells) from 965 blastocysts. Two hundred and thirty-two couples produced the blastocysts at an average female age of 36.85 (range 24–50) years. Biopsied blastocyst samples for genomic DNA extraction were collected from patients who underwent maternal genetic tests in the An-An Women and Children Clinic. Patients signed the consent forms and agreed to donate any remaining sample for research. Furthermore, WGS data (20 individuals) and genomic DNA (17 individuals) were obtained from the Taiwan Biobank (TWB) with the approval from the respective ethics committees of the Academia Sinica and the TWB (Wei *et al.*, 2021). Additional control DNAs with known genomic alterations (i.e. NG16944, NA00857, and NA04375) were purchased (Coriell Institute for Medical Research, Camden, NJ, USA) and used as standards for assay development.

We transferred 216 blastocysts, and the criteria to define ‘pregnant’ and ‘non-pregnant’ were as follows. In the case of single embryo transfer (SET), a ‘pregnant’ or ‘non-pregnant’ event was called when one or no sac was observed, respectively. A ‘pregnant’ or ‘non-pregnant’ event was called for a multiple transfer event when the number of observed sacs matched the number of transferred embryos or no sac was observed, respectively. Any cases showing discrepancies between the number of transferred embryos and the number of observed sacs were removed from further analysis. Among the 153 cycles of frozen-thawed embryo transfer (FET), 69 (45.1%) and 84 (54.9%) were classified as pregnant and non-pregnant, respectively.

### Patient preparations for egg retrieval and PGT-A

Patients underwent IVF–PGT-A treatment because they had previously experienced RMs and/or infertility in the setting of advanced maternal age. For controlled ovarian stimulation, patients were treated with an antagonist protocol with hMG: (Menopur^®^, Ferring Pharmaceuticals Inc., Parsippany, NJ, USA) or Follitropin alfa/Lutropin alfa (Pergoveris Merck Serono, Modugno, Italy) and a GnRH antagonist (0.25 mg, Cetrorelix; Merck Serono, Modugno, Italy). The follicle stimulation hormone products were usually started within the first 2–3 days after the period begins, with a starting dose between 150 and 300 IU per day. The dosage was adjusted as the stimulation progressed. hCG (Ovidrel, Merck Serono, Modugno, Italy) was injected at a dose of 250 mcg to induce final oocyte maturation when at least two dominant follicles reached a diameter of >17 mm. Eggs were retrieved via transvaginal ultrasound at 34–36 h after hCG administration.

### Fertilization, embryo culture, and embryo biopsy

ICSI was performed following the removal of cumulus cells, and the presence of two pronuclei and two polar bodies 16–18 h after injection was ascertained as normal fertilization. Fertilized embryos were cultured in sequential media to the blastocyst stage. On Day 3, a laser-created narrow channel was made in the zona pellucida of all embryos. On Day 5, fully differentiated embryos were biopsied using suction to gently extrude and pull 3–8 TE cells from blastocysts. The biopsied cells were washed in 1× PBS and collected into a microcentrifuge tube with PBS. The cells were used directly for whole genome amplification (WGA).

### WGA and NGS

Following the manufacturer’s instructions, WGA was performed using the REPLI-g Single cell kit (QIAGEN, Hilden, Germany). Qubit dsDNA HS assay kits examined the DNA concentrations with a Qubit fluorometer (Thermo Fisher Scientific, Waltham, MA, USA). Approximately 1 µg of WGA DNA was used for library construction using the Ion Xpress Plus gDNA Fragment Library Preparation Kit Set (Thermo Fisher Scientific). Template positive Ion sphere Particles (ISPs) were generated using Ion 200 Hi-Q Template kits (Thermo Fisher Scientific) with the Ion OneTouch 2 instrument (Thermo Fisher Scientific) and then enriched with the Ion OneTouch ES instrument (Thermo Fisher Scientific). Sequencing was performed on the Ion Proton™ Sequencer (Thermo Fisher Scientific) using an Ion 200 Hi-Q sequencing kit.

### Bioinformatics flowchart

An in-house pipeline was developed and established to analyze and report ploidy for each examined embryo. Briefly, unique reads from ∼5 million sequencing reads generated for each library were mapped to the hg19 human reference genome using the Torrent Mapping Alignment Program (TMAP, https://github.com/iontorrent/TS/tree/master/Analysis/TMAP). After alignment, unmapped reads, duplicate reads, reads with low mapping scores, and reads with greater than one mismatch with the reference genome were removed using BEDtools (https://bedtools.readthedocs.io/en/latest/). The reference genome was divided into non-overlapping bins (1 Mb region), and the number of reads mapped to each bin was counted. Samples were then normalized by summed total coverage to 1; regions with no reads were replaced by 0.0000001. The ratio of regional coverages was converted to the chromosomal CN. The CN in euploid embryos was 2; an aneuploidy is called when >10 Mb shows 80% copy gain (i.e. 2.8 in autosomes and 1.8 in sex chromosome) or loss (i.e. 1.2 in autosomes and 0.2 in sex chromosome).

### Relative CN of mtDNA estimation

After read alignment, genomeCoverageBed files were generated using BEDTools, and the fraction of total sequenced bases aligned to the mitochondrial genome was used to calculate the CN of mtDNA (mtCN). The relative mtCN was calculated using a previously published method (Ding *et al.*, 2014). Briefly, reads of mtDNA in each embryo were first calculated as a percentage and coverage, then converted to the mtCN. The formula CN = CL/2TL was used, where CL is the coverage of mtDNA divided by the length of mtDNA, and the TL is the coverage of chromosomes (22, +XY) divided by the length of chromosomes (22, +XY).

### TL estimation by quantitative PCR and NGS

According to the manufacturer's instructions, the TL measurement was performed using the Absolute Human Telomere Length Quantification qPCR Assay Kit (AHTLQ, ScienCell, Carlsbad, CA, USA). The AHTLQ kit is designed to measure the average TL of human cells directly. The telomere primer and single copy reference primer were designed to recognize and amplify telomere sequences and a 100-bp-long region on human chromosome 17, respectively. In addition, the reference genomic DNA sample with known TL serves as a reference for calculating the TL of target samples.

A digital estimation of TL using WGS data (BAMs) was performed using the TelSeq tool (Ding *et al.*, 2014). Telseq (https://github.com/zd1/telseq) estimates TL from the calculated number of telomeric repeats TTAGGG/CCCTAA presented in each read. We defined reads as telomeric if they contained *k* (default threshold value of *k* = 7) TTAGGG repeats. These can then be translated into an estimate of the physical length via a size factor ‘*s*’ and a constant length ‘*c*’ in *l* = *tksc*, where *l* is the length estimate, *tk* is the abundance of telomeric reads at threshold *k*, and *c* is a constant for genome length divided by the number of telomere ends 46 (23 × 2).

### Statistical analysis

Data were analyzed using paired or unpaired Student’s *t*-tests, one-way ANOVA followed by Tukey’s multiple tests, and linear and logistic regression analysis, all using GraphPad Prism 6.0 (GraphPad Software, La Jolla, CA, USA). All data were presented as mean ± SE, and the significance was set at *P* < 0.05. Parameters that were compared during this study included maternal age (<34 years old or ≥34 years old), embryo chromosome status (euploid vs aneuploid), and embryo implantation potential (implantation and no implantation). Multiple logistic regression analysis assessed the association between the risk factors and IVF outcome. The principal model included the following candidate variables: maternal age, family history of chromosome abnormality, history of RM, individual embryo mtCN, and TL.

### Parameter setting for the tree and random forest models

The tree model was performed using Recursive Partitioning and Regression Trees (rpart) implemented in the R package (https://www.R-project.org/). To avoid overfitting the data, we applied the combinations of minsplit (=25) and minbucket (=10) parameters in rpart to grow a succinct tree with clear decision rules. The minimum node size for further splitting is 25 observations, and each terminal node (leaf) has at least 10 observations.

The random forest (RF) model was performed using randomForest implemented in the R package. A grid search was used to find the values for three significant parameters, ntree (number of trees to grow), mtry (number of variables randomly sampled as candidates at each split), and nodesize (minimum size of terminal nodes). The selection criteria are looking for higher test accuracy and lower standard deviation. The final setting of the parameters is (ntree, mtry, nodesize) = (500, 2, 8). Five-fold cross-validation has been applied as a criterion with the full (148) and restricted (130) euploid embryos to evaluate the prediction performance and stability of the statistical models.

## Results

### Aneuploidy detection using an in-house developed pipeline

To assess the ploidy of each embryo, an in-house established WGS-based workflow of PGT-A, summarized in Supplementary Fig. S1, was applied. The workflow implemented wet lab (from DNA extraction to high throughput sequencing) and dry lab (from NGS data processing to chromosome aneuploidy report) and was executed in one laboratory. The overall procedures for running WGS-based PGT-A workflow take <1 day and only require 4 h for the hands-on experiment.

A total of 965 blastocysts from 232 cycles (164 patients, ages ranging from 24 to 50, median age 37 years) were used in this study (Table 1). Between 5 and 10 TE cells were biopsied from each embryo and underwent WGA. Among them, 879 WGA products have been followed by the WGS workflow for aneuploidy detection, and embryo classification as euploid or aneuploid was based on results obtained after NGS analysis. Of these, 473 (53.8%) were characterized as being chromosomally normal, while the remaining 406 were determined to be aneuploid (46.2%). As shown in Table 1, the percentage of aneuploid embryos was 26.5% in the young maternal age (<34 years old) group; it increased significantly (*P* < 0.001) to 53.5% in the advanced maternal age (≥34 years old) group. The data, therefore, support the notion that advanced maternal age is highly associated with aneuploid embryos.

**Table 1.** Next-generation sequencing analysis of human blastocysts in patients undergoing IVF with preimplantation genetic testing for aneuploidy.

		Maternal age (years)		
	Total	<34	≥34	*P*-value
Case	164	24–50 (Mean: 36.7, Median: 37)		
Cycles	232	55	177	–
WGA embryos	965	187	778	–
WGS embryos	879	238	641	<0.001
Euploid	473	175	298	
Aneuploid	406	63	343	
Transferred embryos	216	71	145	0.002
CA	33	10	23	–
RM	31	2	29	–
Others	152	59	93	–

		**Pregnant**	**Non-pregnant**	

Tracked embryos	153	69	84	0.002
CA	21	3	18	–
RM	25	9	16	–
Others	107	57	50	–

WGA, whole genome amplification; WGS, whole genome sequencing; CA, chromosome abnormality; RM, recurrent miscarriage.

Two hundred and sixteen of the normal blastocysts that underwent uterine transfer were followed. We divided the transferred embryos into three groups: embryos from a woman with a family history of a chromosome abnormality (CA, 33 embryos, 15.3%), a woman with a history of RM (31 embryos, 14.4%), and women without these two defined conditions (152 embryos, 70.3%). Among them, a disproportionate number of embryos coming from young versus advanced maternal age groups was observed (*P* < 0.01). The fact that almost all embryos from women with a history of RM are in the advanced maternal age group (93.5%) implies that RM may be the primary determinant for women in the advanced maternal age groups in the IVF center. Furthermore, among 153 transferred embryos that can determine pregnancy outcomes, we found that embryos from women with a family history of CA showed the lowest pregnancy rate (3 out of 21 embryos, 14.3%), followed by women with a history of RM (9 out of 25 embryos, 36%), and women without these two defined conditions had the highest pregnancy rate of 53.3% (57 out of 107 embryos). The data indicate that other than advanced maternal age, factors like parental family history of CA and maternal pathophysiology conditions may also impact the pregnancy outcome following FET.

### mtDNA CN is a biomarker for embryo aneuploidy

**Figure 1.**
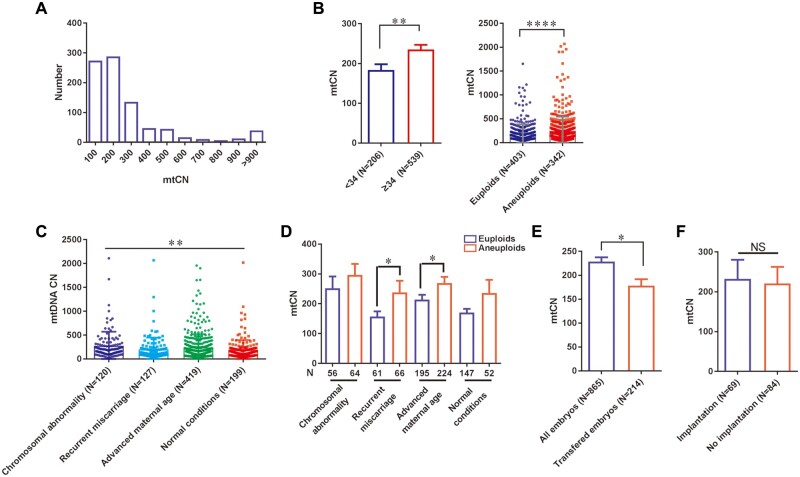
**Mitochondrial DNA copy number is a biomarker for human embryo aneuploidy but not implantation potential.** (**A**) The bar chart shows the population of mtCN in all NGS data analyzed in this study. Among them, 95% of samples have mtCN <900. (**B**) Left: The mtCN was sorted by maternal age: <34 years old and ≥34 years old; statistics results showed the ≥34-year-old group has a significantly higher mtCN (*P* = 0.0029). Right: Comparing mtCN in euploid and aneuploid embryos, statistics showed that the aneuploids have significantly higher mtCN (*P* < 0.0001). The difference between means was calculated with unpaired Student’s *t*-test. (**C**) The mtCN estimated from various groups was compared, showing that the estimated mtCN in embryos from couples with different clinical conditions significantly differed (*P* < 0.01). The difference among group means was calculated with one-way ANOVA. (**D**) Further analysis using an unpaired *t*-test showed that mtCN levels in embryos that are euploid and aneuploid are similar in chromosomal abnormality (*P* = 0.206) and Normal (*P* = 0.078) groups, and are significantly different in Miscarriage (*P* = 0.031) and Advanced maternal age (*P* = 0.017) groups. (**E**) The comparison of all embryos and transferred embryos showed a significant decrease in mtCN in transferred embryos (*P* = 0.011). (**F**) mtCN in implantation embryos and no implantation embryos were compared using an unpaired *t*-test and showed no difference between these two groups (*P* = 0.76). NS, not significant, *P < 0.05, **P < 0.01, ***P < 0.001, ****P < 0.0001. mtCN, mitochondrial DNA copy number; NGS, next-generation sequencing.

Previous studies have reported that mtDNA level is critical for embryo development, and an embryo with a higher amount of mtDNA may not be suitable for embryo transfer (Diez-Juan *et al.*, 2015; Fragouli *et al.*, 2015). To study if mtDNA level can also be used as a biomarker for embryonic implantation potential, we calculated the mtDNA percentage using NGS-PGT-A data in each embryo and converted it to mtCN (Ding *et al.*, 2014) in our study population. As shown in Fig. 1A, the estimated mtCN showed a distribution that skews toward a lower level, with the majority of mtCN (>95%) in embryos being less than 800 copies (Fig. 1A).

We then analyzed mtCN in association with several factors and clinical outcomes. In agreement with previous observations, we found a significantly lower mtCN correlated with younger maternal age (183.7 ± 14.45 vs 235.2 ± 11.72, *P* < 0.01, Fig. 1B, left) and euploid embryos (189.2 ± 9.72 vs 251.6 ± 16.79, *P* < 0.0001, Fig. 1B, right). As previous studies did not investigate details of mtCN with different physical/maternal conditions, we analyzed the mtCN in the embryos of each group. Although the overall mtCN varied among different groups (*P* < 0.01, Fig. 1C), we found a consistent trend that higher mtCN is associated with aneuploid embryos in all groups (Fig. 1D), and the differences reached significance in RM and advanced maternal age groups (*P* < 0.05). The results support the notion that mtCN is a biomarker for embryo aneuploidy, regardless of each embryo's physical and/or maternal conditions.

As only euploid embryos are subjected to transfer, there is no surprise that mtCN from the transferred embryos was significantly lower than that of overall mtCN in all assayed embryos (*P* < 0.05, Fig. 1E). We further analyzed mtCN in 153 transferred embryos with known pregnancy outcomes to evaluate the potential of using it as a predictor for implantation. In contrast to the previous studies (Diez-Juan *et al.*, 2015; Fragouli *et al.*, 2015), we found no differences in mtCN between the successful implantation (139.8, n = 69) and implantation failure groups (137.5, n = 84, *P* = 0.76, Fig. 1F). These data indicate that mtCN, although an indicator for embryo aneuploidy, has no role in impacting IVF outcomes after FET in these women.

### TL estimated at the blastocyst stage is highly associated with pregnancy outcome

To evaluate the biological significance of TL in embryo survival after a transfer, we sought to estimate TL in individual embryos using genomic information obtained from PGT-A sequencing reads. We applied a computer program to calculate the presence of different numbers of TTAGGG/CCCTAA repeats in the reads and estimated averaged TL in individual embryos (Ding *et al.*, 2014). The averaged TL was calculated with the number of telomeric repeats from K1 to K7 using the Telseq program, in which the higher *K* value indicates the presence of a more extended telomeric repeat. To assess the program performance, 16 samples with more than full 30× genome coverage available (read count ranges from 745 579 392 to 989 518 652, average = 838 301 931, Illumina system) were obtained from TWB and used to predict TL from individual BAM files (Wei *et al.*, 2021). The averaged TLs estimated from the presence of various telomeric repeats were obtained (Supplementary Table S1). As expected, the abundance of telomeric reads decreased at repeat unit K increased, and so did the predicted TL decline. Interestingly, the mean predicted TL declined dramatically from K1 to K3 and settled from K4 to K7. Finally, we obtained an average predicted TL at K7 of 2.79 ± 0.89 kb using WGS data from TWB, shorter than 7.5 kb estimated using TwinsUK data (Ding *et al.*, 2014).

**Figure 2.**
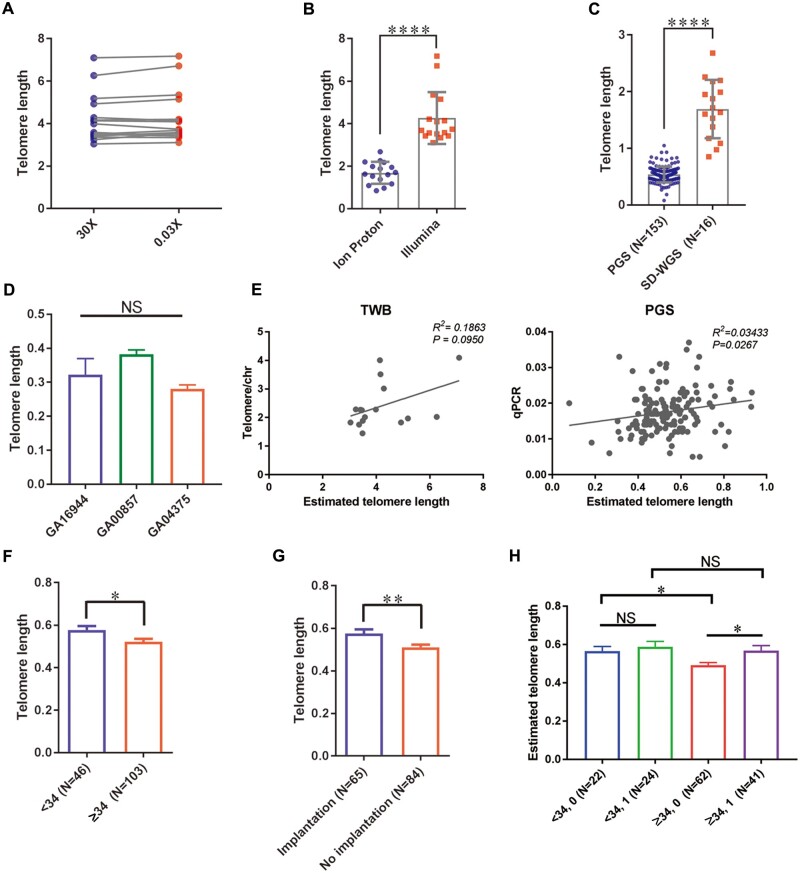
**Digitally estimated telomere length is highly associated with human embryo implantation potential following frozen embryo transfer.** (**A**) The estimated TL from >30×- and 0.3×-coverage PGT-A data showed a high correlation (r = 0.9896), and a paired *t*-test showed no difference between them (*P* = 0.078). (**B**) TL estimation using data from the Ion Proton instrument showed significant differences from the data pattern obtained using Illumina (*P* < 0.001). The difference between means was calculated with an unpaired *t*-test. (**C**) TL was estimated from WGS data generated from DNA samples with and without WGA, and the difference between means was calculated with an unpaired *t*-test and the results showed that the WGA procedure significantly affects TL estimation (*P* < 0.001). (**D**) TL assessments using data generated from three standard cell-line DNAs were compared using two-way ANOVA and showed the estimated TLs from repeated experiments were close and no difference (*P* < 0.001). This indicates that the current procedure is reproducible. (**E**) The digital estimations of cellular and embryonic TLs were compared using Spearman's correlation test and showed a moderate (r = 0.43, *P* = 0.095, left) and weak but significant correlation (r = 0.19, *P* = 0.027, right) to the experimentally measured TLs using qPCR. The data indicate that it is robust and in excellent proximity to its biological TL. (**F**) TL estimated in young-age women ( < 34 years) (0.58±0.02) is longer than that in advanced-age women (>34 years) (0.52±0.014) and shows significant differences between means using an unpaired Student’s *t*-test (*P* = 0.01). (**G**) Digitally estimated TL from 153 women following FET was compared using an unpaired Student’s *t*-test. Results showed that the embryonic TL of women with successful implantation (0.575±0.019) is significantly longer than that of women without implantation (0.511±0.117, *P* = 0.0032). (**H**) Digitally estimated TLs were classified by age ( < 34 and ≥34 years old) and pregnancy outcomes (0: non-pregnant, 1: pregnant) into four groups. Data presented from left to right: young and non-pregnant, young and pregnant, old and non-pregnant, old and pregnant. A significant age-stratified TL effect is shown. NS, not significant, *P < 0.05, **P < 0.01, ***P < 0.001, ****P < 0.0001. TL, telomere length; PGT-A, preimplantation genetic testing for aneuploidy; WGA, whole genome amplification; SR-WGS, shallow-read whole genome sequencing; FET, frozen embryo transfer; qPCR, quantitative PCR; TWB, Taiwan Biobank, PGS, preimplantation genetic screening.

As the current protocol for PGT-A was run under shallow-depth WGS (SD-WGS) conditions with only sequencing at ∼6M reads per sample, we randomly retrieved 6M reads from the original >30× WGS data and performed Telseq to evaluate the effect of read depth on TL estimation (Supplementary Table S1). Given that only <1% of the reads were used, we found the estimated TLs were similar between the original and subsets across all estimations (*P* > 0.05, Fig. 2A), and the phenomenon of predicted TLs decaying from K1 to K3 and settling from K4 to K7 was also observed. In parallel, we experimentally ran SD-WGS using 16 additional genomic DNAs and compared TL estimations between two datasets. While running under similar total read numbers per sample (i.e. 6M reads), TL estimation using an Ion Proton instrument in our system showed a decline from K1 to K7, which is distinct from the data pattern obtained previously (Supplementary Table S2), and the differences are significant (*P* < 0.0001, Fig. 2B and Supplementary Fig. S2). Since the read length generated from the Ion Proton is longer than Illumina, we reason the results may reflect how much information the Telseq program can extract from 200- and 150-bp read length, respectively. Taken together, the results demonstrate that total read count is not a critical factor affecting TL estimation using the Telseq program. Nevertheless, various sequencing platforms that generate sequences with different lengths change TL final approximation.

Next, we took individual BAM files from 153 transferred embryos with known pregnancy outcomes to estimate TL. Averaged TLs of 0.066 ± 0.038 kb were obtained for K7 (the presence of 7 telomeric repeats) using whole genome amplified DNAs followed by SD-WGS, which is shorter than the TL of 0.476 ± 0.184 kb estimated previously using genomic DNA following the same SD-WGS procedure, and the difference is significant (*P* < 0.0001, Fig. 2C and Supplementary Fig. S3). These data showing the WGA procedure that magnifies only a partial of the genome (Biezuner *et al.*, 2021) certainly influence the TL prediction. As such, we subsequently assessed the reproducibility of TL prediction using SD-WGA DNA. Three standard genomic DNAs were run independently three times with the established workflow, followed by NGS data analysis and TL prediction. As shown in Fig. 2D, while TL estimations varied among samples (*P* < 0.05), estimated TLs from repeated measurements were close and showed no difference (*P* = 0.393). The results demonstrate that the digital estimation of TL using whole genome amplified and low-pass WGS data is reproducible.

We validated the digital measurement of TL using quantitative PCR (qPCR). We first established a qPCR assay using TWB DNAs and an average TL of the target genomic DNA sample per diploid cell of 221.21 ± 74.57 kb. The cellular TLs measured by qPCR were similar to the reference TL of 233 ± 10 kb used in the qPCR assay, indicating the assay is valid. Next, the digital-estimated and qPCR-measured TLs were compared and achieved the correlation coefficient of 0.43 (R^2^=0.19, *P* = 0.095, Fig. 2E, left). We further applied qPCR assays in the WGA-DNA from the identical 153 embryos and compared them to the corresponding digital predictions. The mean TL of 1.66 ± 0.62 kb in WGA embryonic DNA is much shorter than the reference TL of 233 ± 10 kb used in the qPCR assay. Nevertheless, a significant positive correlation was obtained between digitally estimated TL and that experimentally measured using qPCR (*r* = 0.19, *R*^2^=0.03, *P* < 0.05, Fig. 2E, right). The positive correlations between digital-estimated and qPCR-measured TL in cellular and WGA DNAs support the notion that Telseq-estimated TL could capture the true biological telomere feature. Furthermore, in agreement with the common notion that TL is inversely associated with age (Benetos *et al.*, 2001), we found TL estimated in young women (0.58 ± 0.02 kb) is longer than that estimated in women of advanced age (0.52 ± 0.014 kb) and the difference is significant (*P* < 0.05, Fig. 2F). The data indicate that the digital estimation of TL is robust and in excellent proximity to its biological TL, despite the TL being estimated from various coverages or partially amplified genomes.

To assess whether TL influenced the ability of an embryo to implant and initiate a pregnancy, we retrospectively analyzed TL data of 153 transferred blastocysts with pregnancy outcomes. Among them, 69 FETs resulted in an ongoing clinical pregnancy, while the remaining 84 embryos failed to implant. The TLs of embryos with successful implantation and implantation failure were compared; a significantly longer TL (0.58 ± 0.019 kb) was found in embryos that successfully implanted compared to those that failed to implant (0.51 ± 0.013 kb, *P* < 0.05, Fig. 2G). Collectively, these data demonstrated that the digital estimation of TL using whole genome amplified and low-pass WGS data is feasible, robust, and reproducible, and a longer TL measured from embryos at blastocyst stage is highly associated with embryo implantation potential.

### TL is the main factor impacting implantation outcome

To identify the significant factors that determine the pregnancy outcome after FET, the association between five factors, including maternal age, family history of CA, history of RM, embryo mtCN, and TL, and implantation outcomes were evaluated individually using multiple logistic regression analysis (Table 2). In addition, odds ratios and 95% CI for all risk factors were calculated and compared in different datasets (Table 2). We found that TL (OR 79.05, 95% CI: 5.01–1247.82), CA (OR 5.91, 95% CI: 1.66–21.03), and RM (OR 2.47, 95% CI: 1.06–5.78) were significant predictors for pregnancy outcome in all participants, while TL (OR 65.78, 95% CI: 2.45–1768.40) and CA (OR 6.72, 95% CI: 1.87–24.19) had a significantly elevated risk for healthy (all participants excluding women with CA and RM) and disease (all healthy women joined by women with CA or RM) participants, respectively. Interestingly, RM was significant in the 5-factor regression model, but the significance dropped when RM alone was examined in the model (Table 2, all participants vs disease). The results suggest that the RM effect uncovered in the full model may be confounded with CA, as most CA patients are indeed listed as RM. In addition, the impact of CA on pregnancy outcome suggests that its influence may be beyond the chromosome integrity *per se.*

**Table 2.** The association of various risk factors in women with pregnancy outcome assessed by logistic regression analysis.

	N	odds ratio	95% CI	*P*-value
All participants				
Age	152	0.975	0.908–1.0482	0.498
mtCN	152	1.000	0.999–1.001	0.542
TL	148	79.054	5.008–1247.824	0.002
CA	152	5.91	1.66–21.03	0.006/0.0025
RM	152	2.47	1.06–5.78	0.037
Healthy				
Age	106	1.003	0.924–1.087	0.952
mtCN	106	1.000	0.999–1.001	0.927
TL	103	65.776	2.447–1768.402	0.013
Disease				
CA	123	6.72	1.87–24.19	0.0012
RM	131	1.99	0.81–4.91	0.13

mtCN, mitochondria copy number; TL, telomere length; CA, chromosome abnormality; RM, recurrent miscarriage. The ‘Healthy’ group includes all participants excluding women with CA and RM. The ‘Disease’ group includes all ‘Healthy’ women and is joined by women with CA or RM.

In agreement with the results from our previous single-factor data analyses, mtCN is not associated, but TL consistently shows a significant association with pregnancy outcomes in all tested models. While the age effect is insignificant as a single factor in our dataset, we stratified age groups and performed statistical analysis to compare the TL effect on pregnancy outcomes in different age groups. Our results revealed no TL differences in pregnancy outcomes in the younger age group (<34 years, blue and green bars, *P* = 0.538, Fig. 2H). At the same time, a significantly longer TL (purple bar, 0.57 ± 0.03 kb) was observed in embryos with successful pregnancy than those with implantation failure (red bar, 0.49 ± 0.01 kb, *P* = 0.0132) in the older (≥34 years) age group. Furthermore, a much shorter TL was detected in the embryos from the advanced maternal-age women (≥34 years, red bar, 0.49 ± 0.01 kb) than the younger women (<34 years, blue bar, 0.57 ± 0.02 kb, *P* = 0.0142) in the implantation failure groups. Interestingly, embryonic TL estimated from the advanced maternal age women (purple bar, 0.57 ± 0.03 kb) was slightly shorter than that estimated from the younger women (green bar, 0.59 ± 0.03 kb) in the successful implantation groups, but the differences are small and not significant (*P* = 0.60).

We further studied the age-adjusted correlation between TL and implantation outcome using a logistic regression model and the coefficients obtained for TL and age were 4.1713 and 0.3498, respectively. The result of the logistic regression suggests that for every TL value, the odds of successful implantation in the younger age group (<34 years) are about 1.42 times (i.e. Exp(0.3498)) larger than that in the older age group (≥34 years). An age-adjusted odds ratio of 1.52 was also obtained (i.e. Exp(4.1713×0.1)) for every 0.1 kb TL increment on successful implantation. Overall, these results revealed the vital role of TL in governing implantation potential. As TLs are similar in pregnant and non-pregnant groups in young women, but TLs in older pregnant women are significantly longer than TLs in older non-pregnant women, the findings support the notion that increasing age is associated with TL shortening. Furthermore, our data also suggest that a minimal TL is necessary to achieve successful implantation.

### A multi-factor model for the prediction of implantation outcome

**Figure 3.**
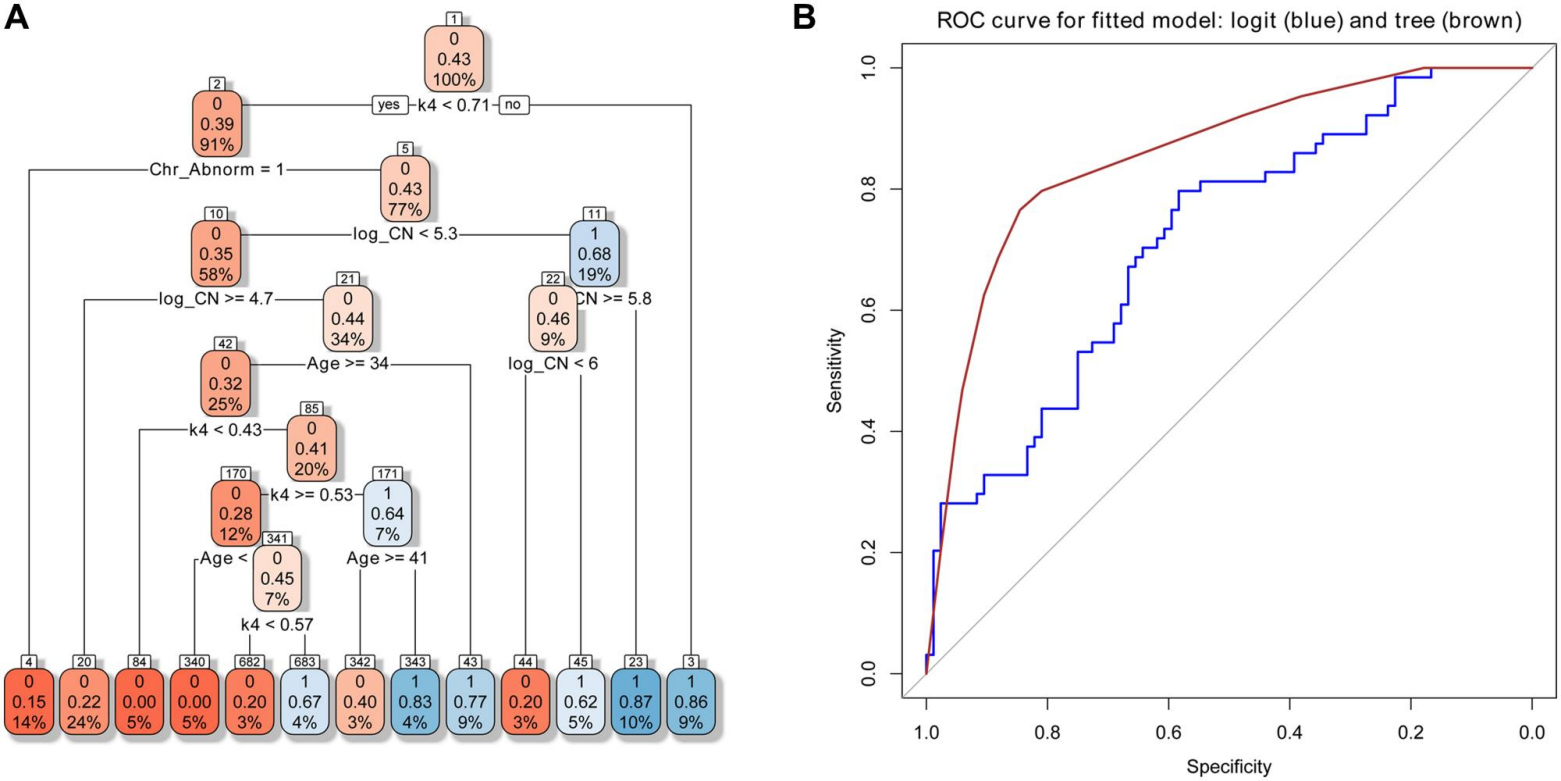
**A fitted tree model for the outcome following frozen embryo transfer. (A)** There are 13 rules (R1 to R13 in the order from right to left) from the tree model listed in the bottom row of the figure. The three numbers inside each node from top to bottom stand for class (1: successful implantation, 0: no implantation), the ratio of successful implantation, and the proportion of sample size. Six rules (with value 1) classify the sample as successful implantation. For example, when K4≥0.71, the tree model will classify a sample as a successful implantation. This rule contains a subset with 9% of the total sample and 86% of the subset with successful implantation. Seven rules (with a value of 0) classify the sample as a non-successful implantation. For example, when K4<0.71 and Chr_Abnorm=1, the tree model will classify a sample as a non-successful implantation. This rule contains a subset with 14% of the total sample and 15% of the subset with successful implantation. **(B)** ROC curves for logistic regression (blue) and tree model (brown) were plotted. The classification accuracies of the logistic regression and tree models are 0.65 and 0.81, respectively. While the sensitivity is the true positive rate, the specificity is the 1− false positive rate. Note that for almost all specificity values, the tree model has higher sensitivity than the conventional logistic regression model. The results support using the tree model over the conventional logistic regression in this study to classify successful implantation. FET, frozen-thawed embryo transfer; ROC, receiver operating characteristic.

Our data revealed a significant age-stratified TL effect and suggested that multiple factors must be considered together to reach the maximum implantation capacity. To further explore the joint effects of these critical factors on successful implantation, we applied three statistical algorithms, including logistic regression, classification tree, and RF, to model fitting using five considered factors and outcome information from transferred euploid embryos. While the conventional logistic regression model for searching linear relations in a classification problem obtained a classification accuracy of 0.65, a tree model with 13 classification rules (Fig. 3A) and an overall classification accuracy of 0.81 was obtained. Among the 13 classification rules, 6 and 7 rules are classified as successful implantation (with value 1) and non-successful implantation (with value 0), respectively. Consistent with the individual factor analysis demonstrated in Table 2, TL is the first independent variable selected by the tree model, which indicates that the parameter has a critical contribution to the classification of implantation outcomes. Our data showed that for a TL≥0.71 (rule no. 1), 86% (12/14) of women are pregnant. On the other hand, when TL is shorter than 0.43 (rule no. 11), 70.8% (17/24) of women failed to become pregnant. Therefore, the multi-factor tree model further supports our theory that a combination of factors, not a single factor, affect the success of implantation. The relative importance of factors was followed by CA, mtCN, and age. Nevertheless, the factor of RM is not selected by the tree model, which may indicate it as a confounding factor with CA, and the impact on successful implantation is minor compared to the other specified factors.

We next compared the sensitivity and specificity between logistic regression and tree models, as shown in receiver operating characteristic (ROC) curves (Fig. 3B). It is clear that the tree model has higher sensitivities over the logistic regression model, given almost all specificities and the AUC of the tree model is 0.86, which is more significant than 0.72 of the logistic regression model. Results from the ROC curve support using the tree model over the conventional logistic regression in this study to classify successful implantation. Furthermore, the confusion matrix of the tree model was constructed (Table 3), and a sensitivity of 0.79 and specificity of 0.82 were obtained in classifying the successful and non-successful implantations, respectively. Furthermore, the full model yields positive predictive values (PPV) and negative predictive values (NPV) of 76.6% and 84.5%, respectively, and an overall accuracy of 81.1% was also achieved.

**Table 3.** The confusion matrix and performance of random forest models using data from the full (148) and restricted (130) euploid embryos.

Model	N	Sensitivity	Specificity	PPV	NPV	F1 score	Accuracy
Full	148						
True positive	49						
True negative	71						
False positive	15						
False negative	13						
Performance		0.790	0.823	0.766	0.845	0.778	0.811
Restricted	130						
True positive	44						
True negative	65						
False positive	9						
False negative	12						
Performance		0.786	0.878	0.830	0.844	0.807	0.838

To evaluate the performance and effectiveness of a machine learning model, a table with four different combinations of predicted and actual values was constructed. The numbers of true positive, false negative, true negative, and false positive were computed from how many times a model made a correct prediction across the entire dataset and were used to calculate clinical statistics, including Sensitivity (the proportion of actual positive cases which are correctly identified), Specificity (the proportion of actual positive cases which are correctly identified), positive predictive value (PPV, the proportion of positive cases that were correctly identified), negative predictive value (NPV, the proportion of negative cases that were correctly identified), F1 score (the harmonic mean of the PPV and Sensitivity), and accuracy (the proportion of the total number of correct predictions that were correct).

The single-tree model has been further explored to build an RF model based on 500 decision trees. To evaluate the prediction performance and stability of the statistical models, we use 5-fold cross-validation as a criterion for the tree- and RF-models (Supplementary Table S3). Generally speaking, the mean training accuracies of the tree and RF models are 0.82 ± 0.02 and 0.91 ± 0.02, indicating that both models classify data with high accuracy. On the other hand, the mean accuracies from test sets of the tree- and RF-models are 0.54 ± 0.05 and 0.61 ± 0.07, respectively; the data may suggest an instability in the prediction accuracy of these models for new data sets.

To improve model performance and achieve more stable prediction precision, a few rules (i.e. R3 and R4) in the tree model that gave poor classification accuracies were removed, and a Target Group was defined if the factor values of the sample were not in the ranges of rules that gave poor classification accuracies. In particular, samples with shorter TL (i.e. K4 < 0.71) and longer mtCN (i.e. log_CN>5.8) were removed, and the restriction resulted in 130 selected euploid embryos in the Target Group. The confusion matrix of the tree model with the Target group was constructed (Table 3), and cross-validation of two statistical models was performed (Supplementary Table S3). While the performances of sensitivity and NPV are the same as the full model, specificity and PPV are improved by 5.5% and 6.4%, respectively. An overall increase of 2.7% in classification accuracy was achieved (Table 3). In addition, while the mean training accuracies of the tree and RF models are 0.82 ± 0.02 and 0.90 ± 0.02, respectively, which are similar to the full sample models, the mean accuracies from test sets of the tree and RF models increased to 0.63 ± 0.10 and 0.71 ± 0.04, respectively (Supplementary Table S3). The data suggest that the RF prediction model will provide a stable prediction for successful implantation with about 75% accuracy in the Target Group.

## Discussion

The current study aimed to provide a solution to prioritizing embryos, based on their implantation potential, in order to reduce a twin pregnancy and the waiting time during pregnancy in an IVF center (Tiitinen, 2019; Fouks and Yogev, 2022). We retrospectively analyzed the digitally estimated TL of each embryo from women who underwent FET after WGS-based PGT-A. Our study has demonstrated that TL is the most prominent factor determining embryo survival and, consequently, a successful pregnancy following FET. The finding is consistent with the vital role TL plays in cell fates (Aksenova and Mirkin, 2019) and embryo persistence (Keefe *et al.*, 2005; Kalmbach *et al.*, 2014; Kohlrausch *et al.*, 2021).

Several factors, including mtCN, have been proposed as important biomarkers for predicting aneuploidy, and implantation or pregnancy potential. In this study, we found biopsied specimens derived from aneuploid embryos contained a significantly greater mtCN than samples from euploid embryos; thus, the results supported a previous finding that mtCN is a good indicator for embryo ploidy and quality (Diez-Juan *et al.*, 2015; Fragouli *et al.*, 2015). While our study demonstrated that mtCN is significantly lower in transferred embryos than in all embryos, mtCN showed no differences between implanted and non-implanted embryos after FET. The result reveals that the elevated mtCN is mainly associated with chromosomal abnormality and suggests that other factor(s) should be involved in the implantation process.

Telomeres are known to control cell division and cellular death; thus playing a critical role in cell maintenance and chromosome integrity (Bernadotte *et al.*, 2016). Previous studies have shown that TL is a biomarker for various human diseases and pathological conditions such as coronary artery disease (Sagris *et al.*, 2022), diabetic kidney disease (Hill *et al.*, 2023), exposure to air pollution (Zizza *et al.*, 2022), and cancers (Holesova *et al.*, 2023). Furthermore, TL is essential in normal development of the embryo and fetus; thus, the abnormal shortening of telomeres is likely involved in embryo loss during early human development (Thilagavathi *et al.*, 2013; Kohlrausch *et al.*, 2021) and is the primary indicator of non-viable fetus elimination (Huleyuk *et al.*, 2018). Although several studies have investigated TLs in various cell types in human, including sperm and polar body, TL estimation directly from a human embryo, and its relation to embryo survival have yet to be studied.

On the other hand, with the increasing amount of NGS data entering clinical routine, TL analyses by short-read NGS have come to the front stage. Although the repetitive nature of telomeric sequences complicates analysis by short-read sequencing, Castle *et al.* (2010) provided a proof of concept to measure telomere content by NGS, and sophisticated bioinformatic tools for WGS-based analysis of telomere content/length have been developed (Ding *et al.*, 2014; Nersisyan and Arakelyan, 2015; Farmery *et al.*, 2018; Feuerbach *et al.*, 2019; Holmes *et al.*, 2022). Despite these tools employing different approaches to identify and quantify telomeric reads, a study used WGS data from a panel of cell strains and cell lines to compare several tools and concluded that WGS-based techniques to determine telomere content offer a robust and accurate alternative to experimental methods such as qPCR (Lee *et al.*, 2017).

The fact that telomeres lengthen during the early cleavage cycles following fertilization through a recombination-based mechanism and that, from the blastocyst stage onwards, telomerase activation in blastocysts just maintains TL during subsequent development (Liu *et al.*, 2007). A study has shown that telomerase expression decreases steadily from a gestational age of 6–11 weeks, and a sharp decline followed by a slow erosion of TL in human fetuses was observed (Cheng *et al.*, 2013). In addition, an early study in measuring TL in humans has demonstrated that TL in blastocysts is significantly longer than at cleavage stages (Turner *et al.*, 2010). These studies reveal that lifetime TL is established at the blastocyst stage and starts erosion beyond that stage. The sharp decline followed by a slow erosion of TL in early embryo development around 6–11 weeks may cause TL to be below the minimum and result in the embryo losing its competence (Kalmbach *et al.*, 2014). These studies led us to hypothesize that embryos with longer TL at the blastocyst stage may have a higher capacity to survive after FET. To test this hypothesis, we established an NGS-based method to estimate TL using low-pass WGS data from the IVF–PGT-A process in the current study. The digital estimation, although affected by read length, is independent of read numbers and is highly reproducible, showing reasonable proximity to the qPCR of TL measurement. In support of our theory, we found that embryo TL is significantly associated with implantation potential following an FET cycle. Taken together, our results demonstrate that the digital estimation of TL using data generated from WGS-based PGT-A is a feasible and accurate approach, and a relatively shorter TL estimated at the blastocyst stage can serve to predict a better implantation potential for that embryo.

To achieve what is the ultimate goal of patients, clinicians and the entire IVF team (i.e. a successful live birth), predictive models which act as a guide for IVF treatment have been developed to help the team perform IVF more effectively and safely. Using advanced computing capabilities, AI-assisted complex predictive models have been developed and applied in various areas of reproductive medicine, ranging from selection of the best embryo for transfer and diagnosis of embryo ploidy, to the prediction of adverse outcomes such as preterm birth and miscarriage (for a review, see Trolice *et al.*, 2021). Among the many diverse models, the AI-assist algorithm has been commonly applied in analyzing image data acquired using traditional (Bormann *et al.*, 2020) or time-lapse (Bori *et al.*, 2020; Berntsen *et al.*, 2022) microscopes to select high-quality embryos. An overall predictive accuracy of 0.7–0.77 was obtained to predict the implantation potential (Bormann *et al.*, 2020). The results show that the prediction sorely based on ‘embryo morphology’ has limitations and point out that other important maternal or embryonic factors still need to be considered simultaneously to achieve higher accuracy. Although complex genomic data are known to provide more critical embryonic information, predictive models have yet to incorporate genomic data from embryos *per se* (i.e. data from PGT-A). As the underlying factors controlling embryo implantation following a FET are multifactorial, many maternal and embryonic features must be considered concurrently to achieve greater accuracy in any successful prediction model.

The limitations of our study include its small sample size. Although we started the investigation with 965 blastocysts from 232 cycles, only 216 blastocysts were transferred. The number was further reduced to 153 blastocysts so that pregnancy outcomes could be accurately traced. Another limitation of this study is that all data were collected from a single IVF center. The uniform and controlled operation of IVF cycles in a single-center thus may cause selection bias. Nevertheless, we present novel findings showing that digitally estimated TL at the blastocyst stage is a reliable predictor of pregnancy potential after a FET cycle.

To the best of our knowledge, this is the first report using the WGS-based method to estimate the TL of embryos and apply it in an AI model to predict the implantation potential of a given embryo. Our study explored embryonic genomics and provided novel findings to show that TL is the most important factor associated with implantation potential after FET. Thus, an AI model using estimated TL accurately predicts the implantation outcome of the given embryo. As elective SET has become the mainstream direction in reproductive medicine, prioritizing embryos based on their implantation potential is crucial for clinical infertility treatment to reduce a twin pregnancy and the waiting time to pregnancy in an IVF center. The AI-powered prediction model established in this study therefore provides a way to improve clinical practice and optimize the chances for people with fertility problems to achieve parenthood.

## Supplementary Material

hoae012_Supplementary_Figures

hoae012_Supplementary_Table_S1

hoae012_Supplementary_Table_S2

hoae012_Supplementary_Table_S3

## Data Availability

The datasets used and analyzed during the current study are available from the corresponding author upon reasonable request.
