# Supplementary material for: Blastocyst telomere length predicts successful implantation after frozen-thawed embryo transfer
Source: Hum Reprod Open. 2024 Feb 24;2024(2):hoae012. doi: 10.1093/hropen/hoae012 (PMC10955253; doi:10.1093/hropen/hoae012)
Supplement: hoae012_Supplementary_Figures [file hoae012_supplementary_figures.docx]

**
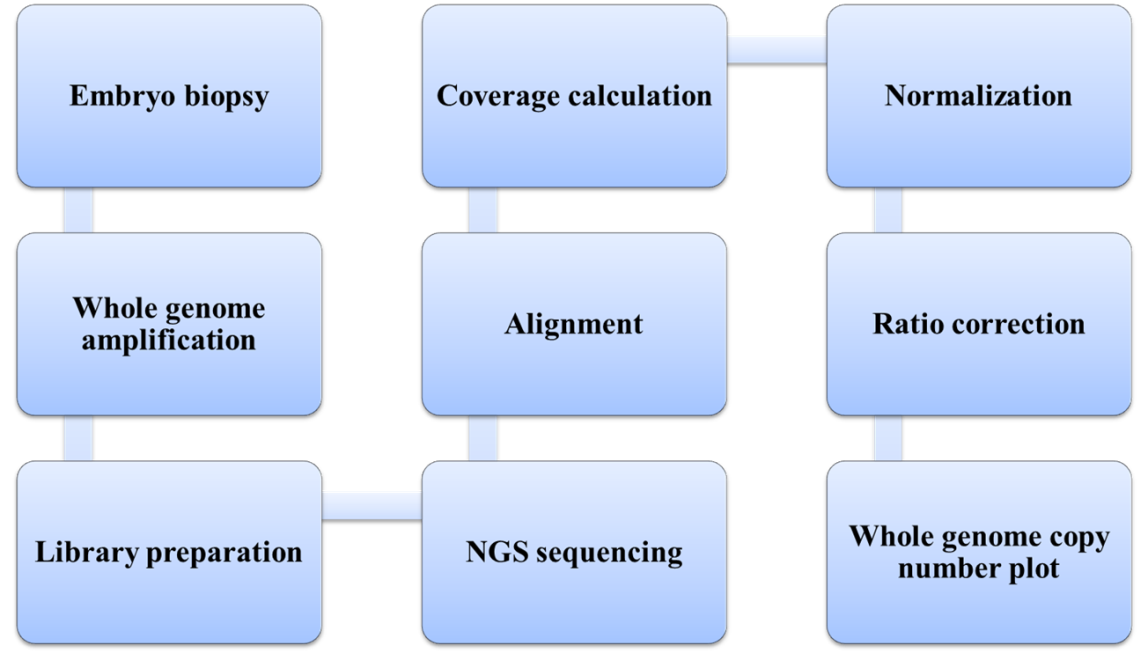
**

**Supplementary Figure S1.** TFDA-approved, WGS-based PGT-A workflow.

We developed a WGS-based workflow in-house to provide clinical PGT-A service. The workflow implemented a wet pipeline (DNA extraction from biopsied TE cells, WGA, library preparation, and NGS sequencing) and dry pipeline (sequence alignment, coverage calculation and normalization, and chromosome aneuploidy report) and was executed in one laboratory. TFDA: Taiwan Food and Drug Administration; PGT-A: Preimplantation Genetic Testing for Aneuploidy; WGA: whole genome amplification; WGS: Whole Genome Sequencing.

**
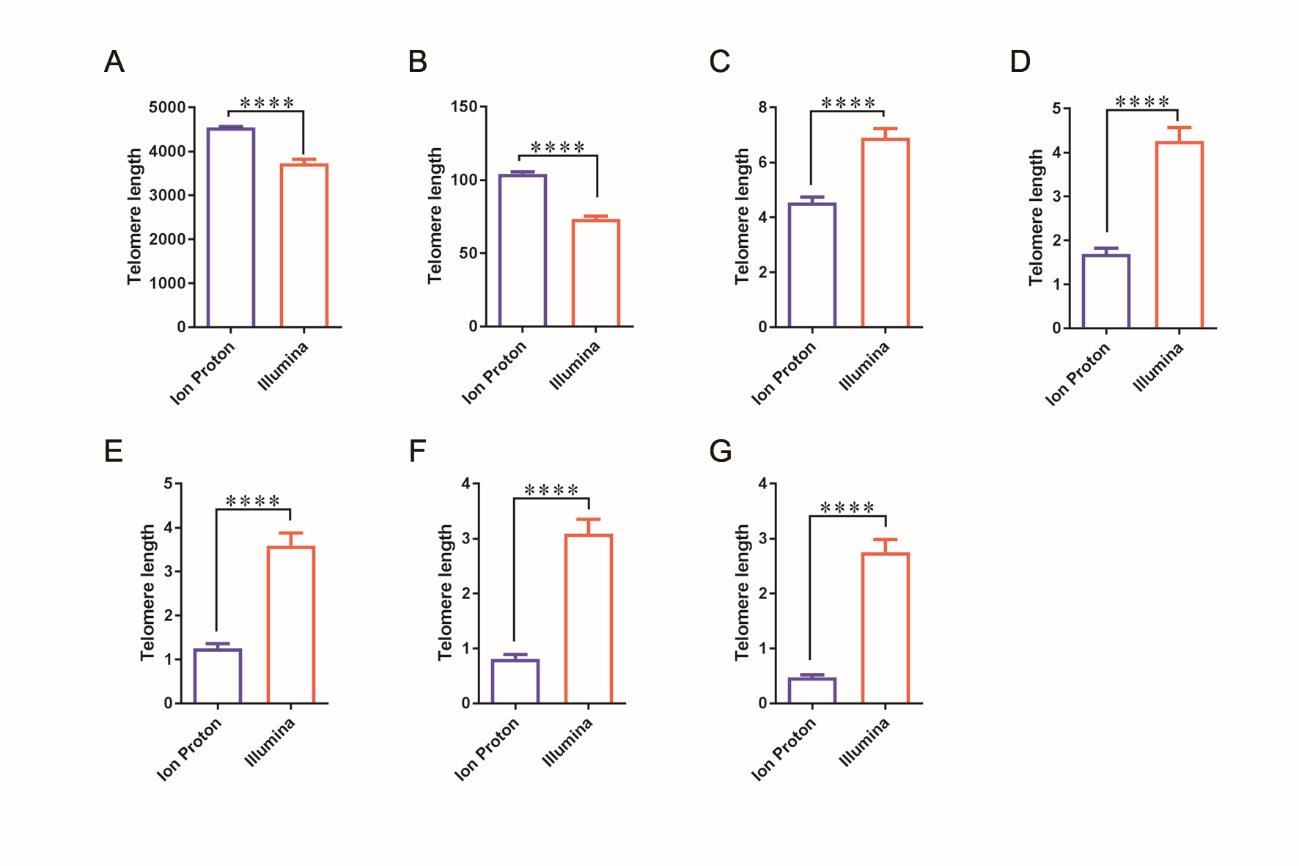
**

**Supplementary Figure S2.** Estimated TL comparisons between PGT-A data generated from the Ion Proton and the Illumina instruments.

While running under similar total read numbers per sample (i.e., 6M reads), TLs using data generated from an Ion Proton instrument are distinct from the data pattern obtained using the Illumina instrument. Comparisons were made based on TL estimation of (A) K1, (B) K2, (C) K3, (D) K4, (E) K5, (F) K6, and (G) K7. The significance of P-values was labeled as * P-value < 0.05, ** P-value < 0.01, *** P-value < 0.001, **** P-value < 0.0001. TL: telomere length; PGT-A: Preimplantation Genetic Testing for Aneuploidy.

**
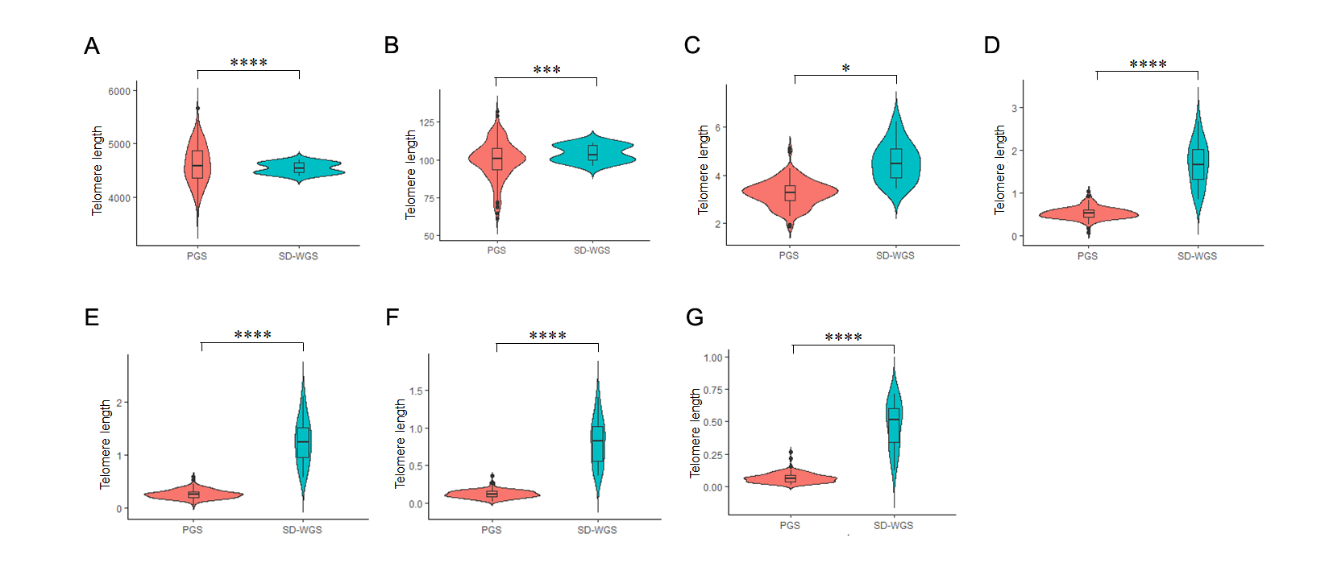
**

**Supplementary Figure S3.** Whole genome amplification procedure affects digital TL estimation.

TLs were estimated from 153 PGT-A data and compared to data generated from genomic DNA following the same SD-WGS procedure. Comparisons were made based on TL estimation of (A) K1, (B) K2, (C) K3, (D) K4, (E) K5, (F) K6, and (G) K7. The significance of P-values was labeled as * P-value < 0.05, ** P-value < 0.01, *** P-value < 0.001, **** P-value < 0.0001. TL: telomere length; PGT-A: Preimplantation Genetic Testing for Aneuploidy; SD-WGS: shallow-read, whole Genome Sequencing; FET: frozen-thawed embryo transfer; qPCR: quantitative polymerase chain reaction.
