# Supplementary material for: Blastocyst telomere length predicts successful implantation after frozen-thawed embryo transfer
Source: Hum Reprod Open. 2024 Feb 24;2024(2):hoae012. doi: 10.1093/hropen/hoae012 (PMC10955253; doi:10.1093/hropen/hoae012)
Supplement: hoae012_Supplementary_Table_S1 [file hoae012_supplementary_table_s1.docx]

**Supplementary Table S1.** The averaged telomere length estimations using whole genome sequencing data with full (>30X) and 6M (0.4X) reads.

|  | **Reads** |  | **K1** |  | **K2** |  | **K3** |  | **K4** |  | **K5** |  | **K6** |  | **K7** |  |
| --- | --- | --- | --- | --- | --- | --- | --- | --- | --- | --- | --- | --- | --- | --- | --- | --- |
| **Sample ID** | Full | 6M | Full | 6M | Full | 6M | Full | 6M | Full | 6M | Full | 6M | Full | 6M | Full | 6M |
| NGS20140601E | 789,051,265 | 6,281,428 | 3608.66 | 3607.21 | 63.33 | 63.39 | 5.81 | 5.52 | 3.58 | 3.43 | 3.01 | 2.91 | 2.61 | 2.63 | 2.32 | 2.47 |
| NGS20140610G | 807,039,965 | 6,423,339 | 3854.53 | 3860.37 | 71.40 | 72.40 | 6.53 | 6.15 | 3.99 | 3.74 | 3.33 | 3.03 | 2.88 | 2.54 | 2.57 | 2.24 |
| NGS20140602C | 816,762,258 | 6,505,619 | 4488.69 | 4485.63 | 83.48 | 84.11 | 7.71 | 7.99 | 4.93 | 5.15 | 4.20 | 4.34 | 3.68 | 3.80 | 3.28 | 3.46 |
| NGS20140703A | 768,324,299 | 6,114,166 | 3266.17 | 3267.72 | 63.88 | 63.62 | 6.75 | 6.81 | 4.16 | 4.21 | 3.49 | 3.59 | 2.99 | 3.15 | 2.67 | 2.80 |
| NGS20140710C | 797,215,264 | 6,347,451 | 3091.54 | 3087.87 | 61.33 | 61.70 | 6.61 | 6.32 | 4.29 | 4.14 | 3.59 | 3.56 | 3.08 | 3.02 | 2.71 | 2.56 |
| NGS2015012C | 812,389,376 | 6,463,617 | 3545.26 | 3550.00 | 69.06 | 68.60 | 6.15 | 6.44 | 3.47 | 3.52 | 2.83 | 2.74 | 2.39 | 2.22 | 2.10 | 1.96 |
| NGS2_20150111H | 745,579,392 | 6,643,490 | 3805.83 | 3803.64 | 78.18 | 80.11 | 8.97 | 9.61 | 6.26 | 6.72 | 5.53 | 5.94 | 4.92 | 5.18 | 4.38 | 4.54 |
| NGS2_20150107G | 943,994,734 | 6,556,244 | 3662.11 | 3686.19 | 76.73 | 76.85 | 7.84 | 7.84 | 5.19 | 5.35 | 4.46 | 4.62 | 3.88 | 4.01 | 3.42 | 3.38 |
| NGS2_20150103F | 936,329,207 | 6,485,101 | 3636.40 | 3621.86 | 76.75 | 76.04 | 9.77 | 9.80 | 7.09 | 7.18 | 6.21 | 6.34 | 5.47 | 5.65 | 4.84 | 5.09 |
| NGS2_20150111B | 939,814,828 | 6,538,869 | 4428.49 | 4425.98 | 89.15 | 90.19 | 6.33 | 6.90 | 3.24 | 3.57 | 2.65 | 2.82 | 2.23 | 2.42 | 1.96 | 2.27 |
| NGS20140601A | 989,518,652 | 6,879,216 | 4133.93 | 4146.93 | 84.36 | 84.02 | 7.28 | 7.55 | 4.14 | 4.09 | 3.42 | 3.33 | 2.92 | 2.78 | 2.59 | 2.49 |
| NGS20150111D | 846,888,698 | 6,736,776 | 3785.83 | 3783.10 | 72.39 | 73.16 | 5.97 | 5.99 | 3.49 | 3.43 | 2.97 | 2.89 | 2.57 | 2.40 | 2.28 | 2.15 |
| NGS2015014G | 798,907,550 | 6,357,059 | 3619.44 | 3629.53 | 69.75 | 70.48 | 5.77 | 5.68 | 3.41 | 3.58 | 2.89 | 3.12 | 2.49 | 2.74 | 2.21 | 2.42 |
| NGS2015018H | 765,492,014 | 6,086,416 | 3692.46 | 3677.04 | 70.36 | 69.60 | 5.48 | 5.32 | 3.04 | 3.11 | 2.52 | 2.63 | 2.16 | 2.24 | 1.89 | 2.03 |
| NGS2015024F | 849,398,079 | 6,759,423 | 3530.67 | 3519.56 | 68.25 | 69.13 | 5.97 | 6.40 | 3.50 | 3.69 | 2.84 | 2.87 | 2.39 | 2.31 | 2.09 | 2.06 |
| NGS2015033G | 806,125,321 | 6,417,645 | 3518.15 | 3526.19 | 68.19 | 69.30 | 5.80 | 6.07 | 3.36 | 3.34 | 2.81 | 2.75 | 2.41 | 2.35 | 2.12 | 2.10 |

To assess the program performance, 16 samples from Taiwan Biobank with more than full 30X genome coverage (columns in gray) and randomly retrieved 6M reads were used to predict telomere length from individual whole genome sequencing data file. The averaged TL in individual samples was estimated using a computer program to calculate the presence of different numbers of telomeric repeats, from K1 to K7, in the sequencing reads, in which the higher K value indicates the presence of a more extended telomeric repeat.
