# Supplementary material for: Blastocyst telomere length predicts successful implantation after frozen-thawed embryo transfer
Source: Hum Reprod Open. 2024 Feb 24;2024(2):hoae012. doi: 10.1093/hropen/hoae012 (PMC10955253; doi:10.1093/hropen/hoae012)
Supplement: hoae012_Supplementary_Table_S2 [file hoae012_supplementary_table_s2.docx]

**Supplementary Table S2.** The averaged telomere length estimations using shallow-depth, whole genome sequencing (~6M) reads.

| **Sample_ID** | **Total_reads** | **K1** | **K2** | **K3** | **K4** | **K5** | **K6** | **K7** |
| --- | --- | --- | --- | --- | --- | --- | --- | --- |
| 170309 | 7,854,604 | 4478.08 | 103.86 | 5.09 | 1.95 | 1.36 | 0.86 | 0.50 |
| 175008 | 6,462,928 | 4440.11 | 97.98 | 5.30 | 2.26 | 1.82 | 1.11 | 0.65 |
| 175009 | 6,286,464 | 4589.30 | 99.75 | 3.45 | 1.17 | 0.95 | 0.75 | 0.50 |
| 175010 | 5,809,453 | 4427.11 | 95.87 | 3.79 | 1.63 | 1.28 | 0.88 | 0.53 |
| 176066 | 6,898,617 | 4663.21 | 109.82 | 5.65 | 2.18 | 1.51 | 1.05 | 0.58 |
| 176068 | 6,559,236 | 4612.50 | 108.87 | 4.72 | 1.99 | 1.53 | 0.96 | 0.57 |
| 176070 | 6,320,256 | 4661.11 | 111.09 | 4.52 | 1.71 | 1.20 | 0.80 | 0.54 |
| 176093 | 6,889,866 | 4484.88 | 101.77 | 3.58 | 0.97 | 0.71 | 0.43 | 0.30 |
| 176081 | 5,775,904 | 4483.16 | 98.40 | 3.57 | 0.85 | 0.58 | 0.36 | 0.13 |
| 176080 | 5,904,970 | 4390.31 | 100.10 | 4.38 | 1.60 | 1.12 | 0.65 | 0.36 |
| 176078 | 5,090,754 | 4472.47 | 102.09 | 4.07 | 1.09 | 0.69 | 0.42 | 0.21 |
| 176026 | 5,814,748 | 4460.31 | 100.40 | 3.93 | 1.38 | 1.04 | 0.58 | 0.36 |
| 176076 | 6,157,252 | 4698.89 | 110.79 | 4.45 | 1.53 | 0.94 | 0.51 | 0.28 |
| 176056 | 6,990,966 | 4636.41 | 109.51 | 5.10 | 2.20 | 1.69 | 1.22 | 0.69 |
| 176162 | 5,719,525 | 4646.24 | 110.91 | 6.23 | 2.68 | 2.10 | 1.41 | 0.71 |
| 176170 | 4,977,086 | 4583.71 | 107.26 | 4.79 | 1.88 | 1.49 | 1.01 | 0.71 |

To assess the program performance, 16 genomic DNA were run with shallow-depth, whole genome sequencing using an Ion Proton instrument, and the averaged TL in individual samples was estimated using a computer program to calculate the presence of different numbers of telomeric repeats, from K1 to K7, in the sequencing reads, in which the higher K value indicates the presence of a more extended telomeric repeat.
