# Supplementary material for: Blastocyst telomere length predicts successful implantation after frozen-thawed embryo transfer
Source: Hum Reprod Open. 2024 Feb 24;2024(2):hoae012. doi: 10.1093/hropen/hoae012 (PMC10955253; doi:10.1093/hropen/hoae012)
Supplement: hoae012_Supplementary_Table_S3 [file hoae012_supplementary_table_s3.docx]

**Supplementary Table S3.** Five-fold cross-validation results of accuracy on the Tree and Random Forest models with data from the full (148) and restricted (130) euploid embryos.

|  | Tree Model | | Random Forest Model | |
| --- | --- | --- | --- | --- |
| Accuracy | Train/Test (148) | Train/Test (130) | Train/Test (148) | Train/Test (130) |
| CV1 | 0.80/0.60 | 0.80/0.69 | 0.90/0.67 | 0.88/0.73 |
| CV2 | 0.80/0.47 | 0.82/0.77 | 0.93/0.63 | 0.93/0.77 |
| CV3 | 0.84/0.53 | 0.86/0.50 | 0.92/0.63 | 0.91/0.69 |
| CV4 | 0.83/0.52 | 0.83/0.58 | 0.90/0.48 | 0.88/0.65 |
| CV5 | 0.82/0.59 | 0.83/0.62 | 0.89/0.62 | 0.89/0.69 |
| Mean | 0.82/0.54 | 0.82/0.63 | 0.91/0.61 | 0.90/0.71 |
| SD | 0.02/0.05 | 0.02/0.10 | 0.02/0.07 | 0.02/0.04 |

To evaluate the prediction performance and stability of the statistical models, we use five-fold cross-validation (CV) as a criterion for the Tree- and Random Forest-models. The five-fold cross-validation is a process in which all data is randomly split into 5 folds, and then the model is trained on the 4 folds, while one fold is left to test a model.
